# *Notes from the Field:* CDC Polio Surge Response to Expanding Outbreaks of Type 2 Circulating Vaccine-Derived Poliovirus — Africa and Philippines, September 2019–March 2020

**DOI:** 10.15585/mmwr.mm6934a6

**Published:** 2020-08-28

**Authors:** Erika Meyer, Neha Sikka, Elias Durry, Deblina Datta

**Affiliations:** ^1^Global Immunization Division, Center for Global Health, CDC; ^2^Icahn School of Medicine at Mt. Sinai, New York City, New York.

In April 2016, a resolution by all members of the 68th World Health Assembly* in coordination with the Global Polio Eradication Initiative (GPEI) resulted in the removal of the Sabin-strain type 2 oral poliovirus vaccine (OPV) component from all immunization activities to avert outbreaks of type 2 circulating vaccine-derived poliovirus (cVDPV2). In the first quarter of 2016, house-to-house supplementary immunization activities (SIAs) with trivalent OPV (containing Sabin-strain types 1, 2 and 3) were conducted in 42 at-risk countries^†^ in an effort to close type 2 immunity gaps in countries with chronically weak routine childhood immunization systems. However, the quality of SIAs in some countries was inadequate, and pockets of unimmunized and underimmunized children remained. Sabin-strain monovalent OPV type 2 (mOPV2) was then successfully used in response to many cVDPV2 outbreaks; however, some outbreaks in sub-Saharan Africa were not promptly controlled and spread to other countries. Where mOPV2 SIA quality was low, prolonged Sabin-strain type 2 circulation allowed new cVDPV2 outbreaks to emerge (*1*). In 2019, 358 cVDPV2 cases were reported, representing a fourfold increase over the 71 cases reported in 2018 and more than tripling the number of countries with outbreaks, from five (*2*) to 16. As of August 2, a total of 236 cVDPV2 cases in 17 countries have been reported in 2020. Among 33 cVDPV outbreaks reported during July 2018–February 2020, 31 (94%) were caused by cVDPV2 (*1*).

To complement CDC’s ongoing technical assistance, the U.S. CDC’s Emergency Operations Center, which activated a Polio Response in December 2011, initiated a “polio surge” in September 2019 to assist country programs. This surge consisted of recruiting CDC volunteers^§^with international experience and skills valuable for outbreak response and then providing several iterations of a 3-day training on surveillance, eradication strategies, SIA preparation and implementation, supportive supervision, and country-specific briefings. Countries were selected to receive surge assistance on the basis of active outbreak or at-risk status, field travel accessibility, and availability of other essential team members such as CDC-supported Field Epidemiology Training Program (FETP) residents and Stop Transmission of Polio (STOP) consultants.^¶^ CDC surge staff members were placed in frontline field positions to strengthen team coordination, planning, and supervision to improve SIA and surveillance quality. Most deployed CDC staff members traveled from duty stations in the United States to countries with active outbreaks across sub-Saharan Africa and in the Philippines; five staff members also supported preparedness efforts in countries deemed to be at high risk for outbreaks because of proximity to outbreak countries (e.g., Namibia, which shares a porous border with Angola). Ultimately, 108 surge staff members deployed to 13 countries** over the course of 6 months (Figure), 12 of which have CDC country offices or staff presence. CDC did not deploy staff members to all countries with active outbreaks because of safety and access issues.

**FIGURE:**
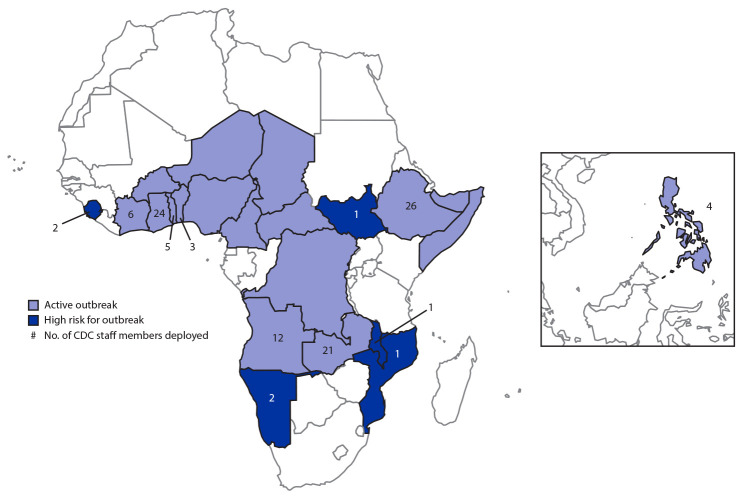
Circulating vaccine-derived poliovirus type 2 outbreak status and number of CDC polio surge staff members deployed in 13 countries, September 2019–March 2020*^,†^ * As of March 11, 2020. ^†^ CDC did not deploy staff members to all countries with active outbreaks because of safety and access issues.

With increasing restrictions on CDC international deployments because of the coronavirus disease 2019 (COVID-19) pandemic, CDC’s Emergency Operations Center first recalled deployed staff members back to CDC country offices in capitals of the supported countries to allow for contingency planning. By March 23 however, all 32 polio surge staff members deployed during March had ended their missions early and returned to the United States. On March 26, the GPEI recommended delaying OPV SIAs until at least June 2020^††^ (*3*). CDC immediately began to identify and contract with additional experienced local FETP or STOP alumni to support polio response activities. With pandemic-prompted limitations in field surveillance and investigations, existing GPEI-supported field staff members redirected substantial time to COVID-19 surveillance and control efforts (*4*).

Disruptions in routine immunization and SIAs because of the COVID-19 pandemic have elevated the risk for increases in vaccine-preventable diseases, including polio (*5*), evident in ongoing confirmations of cVDPV2 spread. Resumption of response mOPV2 SIAs began in late July. When CDC travel restrictions are lifted, allowing mission-critical international travel, polio surge deployments can resume providing technical field support. Although cVDPV2 outbreaks are currently challenging GPEI, progress toward eradication of wild poliovirus has continued; on August 25, 2020, the World Health Organization African Region was certified free of indigenous wild poliovirus transmission, joining the Americas, European, South-East Asia, and Western Pacific regions as wild poliovirus-free.

## Footnotes

* https://apps.who.int/gb/ebwha/pdf_files/WHA68-REC1/A68_R1_REC1-en.pdf#page=15.

† Countries that had achieved <70% routine immunization coverage with the third dose of poliovirus vaccine for at least 1 year during 2010–2015.

§ Sources of volunteers included Epidemic Intelligence Service and Laboratory Leadership Service Officers, returned Peace Corps volunteers, and the Global Rapid Response Team.

¶ These also included national immunization program and World Health Organization staff members in each country.

** Angola, Benin, Côte d’Ivoire, Ethiopia, Ghana, Malawi, Mozambique, Namibia, Philippines, Sierra Leone, South Sudan, Togo, and Zambia.

†† The Polio Oversight Board of the GPEI has since recommended that response SIAs resume as safely and quickly as possible.
